# MicroRNA and Long Non-coding RNA Regulation in Skeletal Muscle From Growth to Old Age Shows Striking Dysregulation of the Callipyge Locus

**DOI:** 10.3389/fgene.2018.00548

**Published:** 2018-11-16

**Authors:** Jasmine Mikovic, Kate Sadler, Lauren Butchart, Sarah Voisin, Frederico Gerlinger-Romero, Paul Della Gatta, Miranda D. Grounds, Séverine Lamon

**Affiliations:** ^1^School of Exercise and Nutrition Sciences, Institute for Physical Activity and Nutrition, Deakin University, Geelong, VIC, Australia; ^2^School of Human Sciences, The University of Western Australia, Perth, WA, Australia; ^3^Institute of Health and Sport, Victoria University, Footscray, VIC, Australia

**Keywords:** aging, miRNAs, lncRNAs, sarcopenia, skeletal muscle

## Abstract

MicroRNAs (miRNAs) undergo high levels of regulation in skeletal muscle development and control skeletal muscle mass, function and metabolism over the lifespan. More recently, the role of long non-coding RNAs (lncRNAs) in skeletal muscle regulation has started to emerge. Following up on our recent study describing the expression pattern and putative roles of 768 miRNAs in the quadriceps muscle of mice at early life stages, we used a high-throughput miRNA qPCR-based array to assess the expression of the same miRNAs in 28-month old male mouse quadriceps muscle. In addition, we report the expression patterns of lncRNAs playing a putative role in muscle development and adaptation from growth to old age. Twelve miRNAs were significantly downregulated in 28-month old muscle when compared with 12-week old muscle. Ten of them clustered at the *Dlk1-Dio3* locus, known as ‘Callipyge,’ which is associated with muscle development and hypertrophy. This collective downregulation was paralleled by decreases in the expression levels of the maternally expressed imprinted LncRNA coding genes *Meg3* and *Rian* stemming from the same chromosomal region. In contrast, the paternally expressed imprinted *Dlk1-Dio3* locus members *Rtl1, Dio3*, and *Dlk1* and the muscle related lncRNAs *lncMyoD1, Neat_v1, Neat_v2*, and *Malat1* underwent significant changes during growth, but their expression levels were not altered past the age of 12 weeks, suggesting roles limited to hyperplasia and early hypertrophy. In conclusion, collective muscle miRNA expression gradually decreases over the lifespan and a cluster of miRNAs and maternally expressed lncRNAs stemming from the Callipyge locus is significantly dysregulated in aging muscle. The *Dlk1-Dio3* locus therefore represents a potential new mechanism for age-related muscle decline.

## Introduction

Maintaining skeletal muscle mass and function is paramount to preserve mobility and whole-body metabolism over the lifespan. Skeletal muscle mass and function naturally declines from maturity to old age (Lexell et al., 1988; Sayer et al., 2013). This occurs as a consequence of the gradual dysregulation of a number of processes that are essential to maintain skeletal muscle structure, metabolism and mitochondrial function (Rooyackers et al., 1996; Short et al., 2005; Birben et al., 2012). Decreased protein synthesis and increased protein degradation are observed in aging humans and rodents (Combaret et al., 2005; Rieu et al., 2006) and lead to a net loss of protein that, in conjunction with a progressive loss of innervation of aging myofibers (Chai et al., 2011), result in age-related muscle atrophy and loss of function (Barns et al., 2014; White et al., 2016b). Muscle regeneration relies on the efficiency of a population of quiescent muscle stem cells, called satellite cells, to activate, proliferate, differentiate and fuse (Yin et al., 2013). Muscle regeneration capacity generally decreases with aging (Yun, 2015), although a central role for intrinsic decline of old satellite cell response is disputed (Grounds, 2014). Elevated Wnt (Fujimaki et al., 2015) and decreased Notch1 (Carey et al., 2007) may contribute to reduced regenerative capacity. Canonical Wnt signaling, essential for satellite cell differentiation and self-renewal (von Maltzahn et al., 2012), inhibits myogenic regeneration and promote muscle progenitor cells to a fibrogenic fate over a myogenic fate (Fujimaki et al., 2015). While the extrinsic age-related mechanisms involved in regeneration are not fully understood, clear alterations are seen in the aging immune system (Jackaman et al., 2013) with a delayed inflammatory response to muscle injury (Grounds, 2014). The aged systemic environment also modifies the extracellular matrix, with a fibrotic environment altering satellite cell behavior and myogenic capacity (Stearns-Reider et al., 2017). Finally, reduced mitochondrial enzyme activity and increased reactive oxygen species levels induce irreversible damage to DNA and other macromolecules, altering cell function (Rooyackers et al., 1996; Short et al., 2005; Birben et al., 2012; Tohma et al., 2014). Hence a wide range of factors collectively contribute to a decline in muscle mass and function with advancing age, termed ‘sarcopenia.’ In the mouse, the onset of sarcopenia occurs between 15 and 18 months of age (Barns et al., 2014; White et al., 2016b) and peaks at 24 months of age (Barns et al., 2014; Soffe et al., 2016).

Muscle homeostasis is fine-tuned by the coordinate action of muscle-specific transcription factors and epigenetic regulators. These include DNA methylation, histone modification (Asp et al., 2011; Howlett and McGee, 2016), as well as the non-coding RNA molecules microRNAs (miRNAs) (Zhao and Srivastava, 2007) and long non-coding RNAs (lncRNAs) (Wang and Chang, 2011), which are the focus of this study. MiRNAs are 20–24 nucleotides single-stranded RNA molecules that make up 1–3% of the eukaryote genome (Zhao and Srivastava, 2007). MiRNAs can be tissue specific and are mostly conserved between species (Kovanda et al., 2014). They regulate gene and protein expression, mostly through degrading messenger RNA (mRNA) or inhibiting protein translation (Bartel, 2004). Skeletal muscle enriched miRNAs, termed myomiRs, include miR-1, miR-133a/b and miR-206 (McCarthy, 2008) and have important and partly redundant functions in regulating satellite cell proliferation, differentiation and apoptosis through fine-tuning Paired Box (PAX) and myogenic regulatory factor (MRF) genes (Huang et al., 2012; Kirby and McCarthy, 2013). However, hundreds of other miRNAs are expressed in skeletal muscle tissue and are subjected to substantial levels of regulation during muscle development (Lamon et al., 2017).

Long non-coding RNAs are classified as non-coding RNA molecules exceeding 200 nucleotides (Engreitz et al., 2016). Like miRNAs, lncRNAs are tissue specific, however, they are poorly conserved between species (Johnsson et al., 2014). It was long thought that lncRNAs lacked any functional roles. Recent findings, however, suggest that lncRNAs have multiple modes of gene regulation and can act as signal, guide, scaffold and decoy molecules for other RNA species, including mRNAs and miRNAs (Wang and Chang, 2011). The role of lncRNAs in skeletal muscle growth (Neguembor et al., 2014) and aging has begun to emerge (Neppl et al., 2017; Zong-Kang et al., 2018). For example, *Chronos* is a lncRNA that increases with age in mouse muscle and impairs skeletal muscle hypertrophy through repressing *Bmp7* (Neppl et al., 2017). The expression of another muscle enriched lncRNA, muscle anabolic regulator 1 (*MAR1)*, also decreases with advancing age in mice and might promote muscle differentiation and regeneration through regulating myogenic mediator *Wnt5a* (Zong-Kang et al., 2018).

We recently observed that a series of lncRNAs, including lncRNA Metastasis associated lunch adenocarcinoma transcript 1 (*Malat1*), the two variants of Nuclear Enriched Abundant Transcript 1 (*Neat1_v1 and Neat1_v2*), long non-coding MyoD (*LncMyoD)* and *Lincmd1* underwent significant dysregulation in early post-natal murine muscle growth (Butchart et al., 2016). *Malat1* and *Neat1* have putative roles in muscle cell differentiation (Sunwoo et al., 2009; Watts et al., 2013; Butchart et al., 2016). Canonical MRF *MyoD* activates *LncMyoD* during myoblast differentiation (Gong et al., 2015), suggesting that *LncMyoD* plays a role in promoting cell cycle exit and commitment to differentiation. This process is fine-tuned by long intergenic non-coding *Lincmd1*, which regulates the timing of myoblast differentiation (Cesana et al., 2011), while also acting as an endogenous sponge for the canonical myomiR miR-133 (Cesana et al., 2011). Research as to whether lncRNAs plays a direct role in facilitating age-related muscle decline is, however, rudimentary.

This study follows on from our recently published work that identified collective miRNA (Lamon et al., 2017) and LncRNA (Butchart et al., 2016) expression in mouse quadriceps muscle over the first 12 weeks of age. This study in young growing mice (up to 3 months) has expanded to analyze muscle from very old mice (aged 28 months old), and examines the expression of 768 miRNAs and selected lncRNAs in mouse quadriceps muscle. Comparing miRNA and LncRNA expression patterns in muscle tissue between such young and old stages of the lifespan is important to increase our understanding of the biological roles of these complex molecules in age-related loss of muscle mass and function, with possible identification of novel therapeutic targets.

## Materials and Methods

### Mouse Muscle Samples

All animals (male C57BL/6J) were purchased from the Animal Resource Centre, Murdoch, Western Australia. Animal experiments were approved by the Animal Ethics committees at the University of Western Australia (RA/3/100/1436); La Trobe University (AEC15-32); and Deakin University (G03-2015). Animal experiments were conducted in strict accordance with guidelines outlined in the National Health and Medical Research Council Code of practice for the care and use of animals for scientific purposes (2004), and the Animal Welfare act of Western Australia (2002). All mice were housed under standard humidity, temperature and lighting conditions, and had *ad libitum* access to food and drinking water. Mice sacrificed at 2 days (*n* = 4), 2, 4, and 12 weeks of age (*n* = 6 per age group) were housed at the University of Western Australia pre-clinical facility as described previously (Lamon et al., 2017). Mice sacrificed at 28 months of age (*n* = 11) were housed at La Trobe University animal facility and sacrificed by decapitation. Quadriceps muscles were dissected out and snap frozen in liquid nitrogen before being stored at -80°C.

### RNA Extraction and Reverse Transcription

Total RNA was extracted from the quadriceps muscle as previously described (Lamon et al., 2017). Total RNA was treated with DNase I Amplification Grade (Thermo Fisher Scientific, Waltham, MA, United States) and total RNA concentration was assessed using the NanoDrop 1000 Spectrophotometer (Thermo Fisher Scientific). For miRNA analysis, RNA (500 ng) was reversed transcribed using the TaqMan MicroRNA Reverse Transcription (RT) kit and Megaplex RT Primers, Rodent Pool A and Pool B v3.0 (Thermo Fisher Scientific) as previously described (Lamon et al., 2017). For mRNA and lncRNA expression analysis, first-strand cDNA was generated was generated from RNA (500 ng) using the High Capacity cDNA Reverse Transcription Kit (Thermo Fisher Scientific) as previously described (Lamon et al., 2017).

### Single-Strand DNA Quantification

First-strand cDNA was generated as described above. cDNA was then treated with RNase H (Thermo Fisher Scientific) according to the manufacturer’s protocol. Single-strand DNA was quantified using the Quant it OliGreen ssDNA Assay Kit (Thermo Fisher Scientific) according to the manufacturer’s instructions and used for mRNA and lncRNA PCR normalization.

### MiRNA Screening and Target Analysis

MicroRNA expression was assessed using the TaqMan Array Rodent MicroRNA A + B Cards v3.0 (Thermo Fisher Scientific) as described previously (Zacharewicz et al., 2014; Lamon et al., 2017). The results from the Megaplex were analyzed using the ExpressionSuite Software v1.0 (Thermo Fisher Scientific) and the data were normalized using the global normalization function, which we previously identified as the technique that most accurately represents biological variations (Zacharewicz et al., 2014; Lamon et al., 2017; Russell et al., 2017). Ct values were transformed into arbitrary units (AU) using the following equation: AU = (1/2)^Ct^10^10^ and expressed relative to the mean value of the latest time point (28 months). Target analysis was performed as described previously (Zacharewicz et al., 2014; Lamon et al., 2017).

### Real-Time PCR

Real-time PCR was carried out using a Stratagene MX3000 thermal cycler (Thermo Fisher Scientific). mRNA and lncRNA levels were measured using 1× SYBR^®^ Green PCR Master Mix (Thermo Fisher Scientific) and 6.25 ng of cDNA. All primers were used at a final concentration of 300 nM, with the exception of Qiagen QuantiTect (QT) primers that were used according to the manufacturer instructions. The PCR conditions have been previously described for mRNAs (Lamon et al., 2017) and lncRNAs (Butchart et al., 2016), with specific melting temperatures shown in Table 1. mRNA and lncRNA levels were normalized to total cDNA input. Primer sequences and characteristics are presented in Table 1 and in Lamon et al. (2017).

**Table 1:** Mouse primers for real time PCR analysis.

Gene	GenBank accession #	Forward primer (5′–3′)	Reverse primer (5′–3′)	Melting temp
*Rtl1*	NM_184109.2	TACTGCTCTTGGTGAGAGTGGACCC	GGAGCCACTTCATGCCTAAGACGA	57°C
*Dlk1*	NM_010052.5	TACCCCTAACCCATGCGAGA	GCACAGACACTCGAAGCTCA	55°C
*Dio3*	NM_172119.2	CCACGTGCAAATGCTCCAAA	TCAGTTCGAGCCACAGCAAT	55°C
*Mef2a*	NM_001033713.2	GCGGAGACTCGGAATTGCAT	GGCTGCCGTTGAAATTGTCT	55°C

**LncRNA**	**GenBank accession #**	**Forward primer (5′–3′)**	**Reverse primer (5′–3′)**	**Melting temp**

*LncMyoD*	N/A	GCAAGAAAACCACAGAGGAGG	GTGAAGTCCTTGGAGTTTGAG	60°C
*Neat1_v1* (short)	NR_003513.3	ACCCTTTTTCATGGGGGTAG	GCTGGATGGAGGCTTGTTTA	55°C
*Neat1_v2* (long)	NR_131212.1	GCTCTGGGACCTTCGTGACTCT	CTGCCTTGGCTTGGAAATGTAA	55°C
*Malat1*	NR_002847.3	ATGTCTCCATGGGGAATGAG	TATGCAGCTTTTCATCAGTAGGA	60°C
*Lincmd1*	NR_131249.1	CTGAAGGACACAAGGTGGCTT	AACTGAGGCTCCCAGTAAGA	60°C
*AK050713*	N/A	TGTTAGGTGCCTTCTCTGCGTGC	GCTGACGCTTCCCTGACAATCTTGTAG	55°C

**LncRNA**	**GenBank accession #**	**Company (catalog number)**	**Melting temp**	

*Rian1 (Meg8)*	NR_028261, XM_901568, XM_922645	Qiagen (QT01197784)	55°C	
*Gtl2 (Meg3)*	NM_144513, NR_003633, NR_027651, NR_027652,		Qiagen (QT00161658)	55°C
	NR_046475, XM_981786, XR_035483, XR_035484			
*Mirg (Meg9)*	XM_488655		Qiagen (QT00285747)	55°C


### Protein Extraction and Western Blotting

Total protein was extracted using RIPA buffer (Millipore, North Ryde, NSW, Australia) with 10 μL/mL Phosphatase and Protease Inhibitor Cocktail (Thermo Fisher Scientific). Total protein content was determined using the BCA Protein Assay Kit (Pierce Biotechnology, Rockford, IL, United States) according to the manufacturer’s instructions. The proteins were separated out using a 4–15% gradient Criterion TGX (tris-glycine) Stain Free gel (Bio-Rad, Hercules, CA, United States) and transferred to a PVDF membrane. The membranes were blocked in 5% skim milk in TBST for 1h at room temperature, after which they were incubated overnight at 4°C with a DIO3 antibody (Biorbyt, 213851) diluted in 5% BSA in TBST at 1:500. Following overnight incubation, the membranes were washed in TBST and incubated for 1 h at room temperature with an anti-rabbit IgG antibody labeled with an infrared fluorescent 800 nm dye (Alexa Fluor^®^ 800; Thermo Fisher Scientific) diluted 1:5000 in 5% BSA in TBST. After washing, the proteins were exposed on an Odyssey^®^ CLx Infrared Imaging System (LI-COR Biosciences) and individual protein band optical densities were determined using the Odyssey^®^ Infrared Imaging System software. All blots were normalized against total protein load using the Bio-Rad Image Lab software (v6.0).

### Statistical Analysis

All data are reported as mean ± SEM. Diagnostic plots of residuals and fitted values were checked to ensure homogeneity of variance and unpaired Student’s t-tests or one-way analysis of variance (ANOVA) were used to compare group means (GenStat v18). Adjustment for multiple testing was performed by using the Bonferroni correction that allows controlling the overall false-positive rate for all inferences and is recognized as the most stringent when the various tests being performed are not highly correlated. The significance level was set at *p* < 0.05.

## Results

### Collective Expression Patterns of miRNAs in Aging Quadriceps Muscle

**FIGURE 1:**
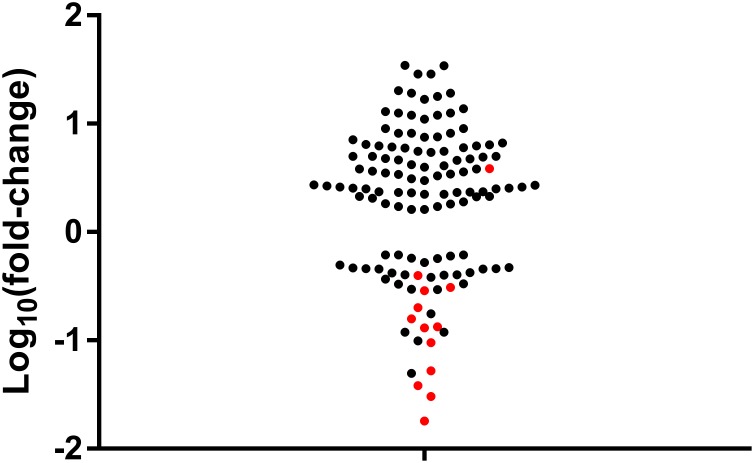
Fold-change of miRNA expression levels in young (12-week) compared with old (28-month) quadriceps muscles of C57Bl/6J male mice. Log_10_ (fold-change of miRNA expression with age). Positive values denote an increase in miRNA expression with age. Negative values denote a decrease in miRNA expression with age. Red dots represent the miRNAs that were returned significant after Bonferroni adjustment.

Our first investigation of miRNA expression patterns with aging compared young adult aged 12 weeks with geriatric 28-month male C57Bl/6J mice. The cut-off for the relevant level of expression of each miRNA was set at Mean (Ct) <32 for each time point, as recommended by the manufacturer. Out of the 768 miRNAs measured, 230 (30%) were expressed at one time point at least (12 weeks or 28 months). A total of 101 miRNAs returned a *p*-value of *p* < 0.05 before adjustment for multiple comparisons (Supplementary File S1). To assess whether a specific trend in expression change was associated with aging, preliminary analysis was conducted on this set of 101 miRNAs. Fold-change was calculated as the ratio of normalized miRNA expression level at the two time points. Overall, 42 miRNAs were downregulated and 59 miRNAs were upregulated at 28 months of age (Figure 1), confirming that individual miRNA expression can be positively or negatively affected by aging.

### Changes in Individual miRNAs in Aging Quadriceps Muscle

**FIGURE 2:**
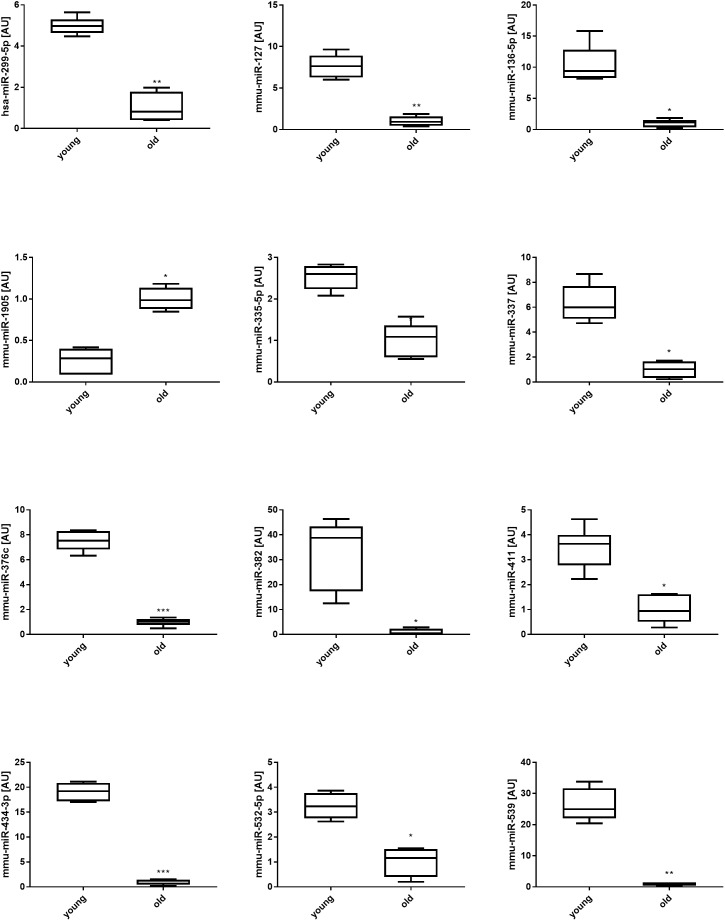
Expression levels of individual miRNAs in young (12-week) compared with old (28-month) muscles. Individual miRNA expression levels are shown for 12 miRNAs that were significantly different in 28-month, when compared with 12-week, old mouse muscle. The data are reported as Mean ± SEM. ^∗^*p* < 0.05; ^∗∗^*p* < 0.01; and ^∗∗∗^*p* < 0.001.

**FIGURE 3:**
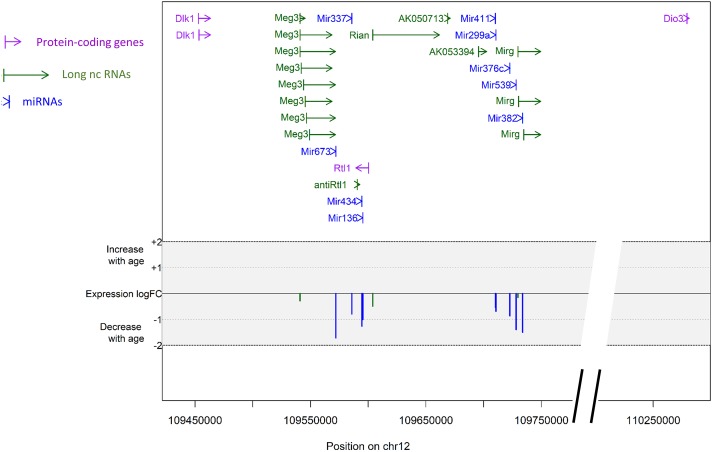
Genomic context of the differentially expressed mRNAs, miRNAs, and lncRNAs in young (12-week) and old (28-month) muscle. We used the basic annotation of GENCODE mouse vM16 to annotate protein-coding genes (mRNAs) (purple), long non-coding genes (lncRNAs) (green), and miRNAs (blue), visible on the top panel. Differential expression between 12-week old and 28-month old mice for a given gene is visible as a vertical bar in the middle panel (log fold-change of expression). All transcripts had decreased expression with age. There was no difference in mRNA expression for the genes *Dlk1, Rtl1*, and *Dio3* between young (12-week) and old (28-month) muscles. This graph was created using an in-house R script. FC, fold change.

To assess individual changes in miRNA expression in 12-week (young) and 28-month (old) quadriceps muscle, *p*-values were adjusted for multiple comparisons using the Bonferroni correction (Supplementary File S1). The expression levels of 12 miRNAs (mmu-miR-434-3p, mmu-miR-376c-3p, mmu-miR-127-3p, hsa-miR-299-5p, mmu-miR-539-5p, mmu-miR-335-5p, mmu-miR-1905, mmu-miR-136-5p, mmu-miR-532-5p, mmu-miR-411-5p, mmu-miR-337-5p, and mmu-miR-382) were significantly different in old when compared with young muscle (Figure 2). All miRNAs, except for mmu-miR-1905, were downregulated with aging, with one additional miRNA, mmu-miR-673-3p, detected only in young (12-week) but not in old (28-month) mouse muscle (data not shown). Ten of these 12 downregulated miRNAs co-localized on two adjacent chromosomic clusters (<30 kb) located on chromosome 12: one cluster containing mmu-miR-127, mmu-miR-136, mmu-miR-337, mmu-miR-434 and mmu-miR-673; and one cluster containing hsa-miR-299-5p, mmu-miR-376c, mmu-miR-382, mmu-miR-539 and mmu-miR-411 (Figure 3). LncRNA encoding genes *Meg3 (Glt2), Mirg (Meg9)*, and *Rian*, and protein encoding genes *Rtl1, Dio3*, and *Dlk1* stem from the same chromosomal region, termed the *Dlk1-Dio3* locus. These genes were therefore selected for further investigation.

### Changes in myomiR Expression in Aging Quadriceps Muscle

**FIGURE 4:**
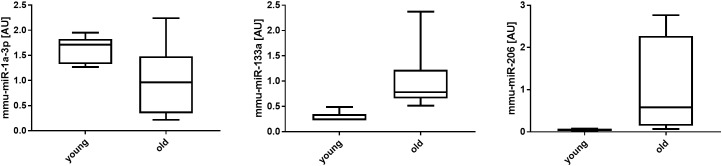
MyomiRs expression levels in young (12-week) compared with old (28-month) muscles. Individual miRNA expression levels are shown for mmu-miR-1-3p, mmu-miR-133a-3p, and mmu-miR-206 in 28-month, when compared with 12-week, old mouse muscle. The data are reported as Mean ± SEM.

The expression levels of the canonical myomiRs, -miR-1-3p, mmu-miR-133a-3p and mmu-miR-206, in 12-week (young) and 28-month (old) muscle are depicted in Figure 4. None of the myomiRs displayed a significant difference in old when compared with young muscle.

### Changes in miRNA and lncRNA Expression Over the Lifespan (Comparison of 5 Ages)

**FIGURE 5:**
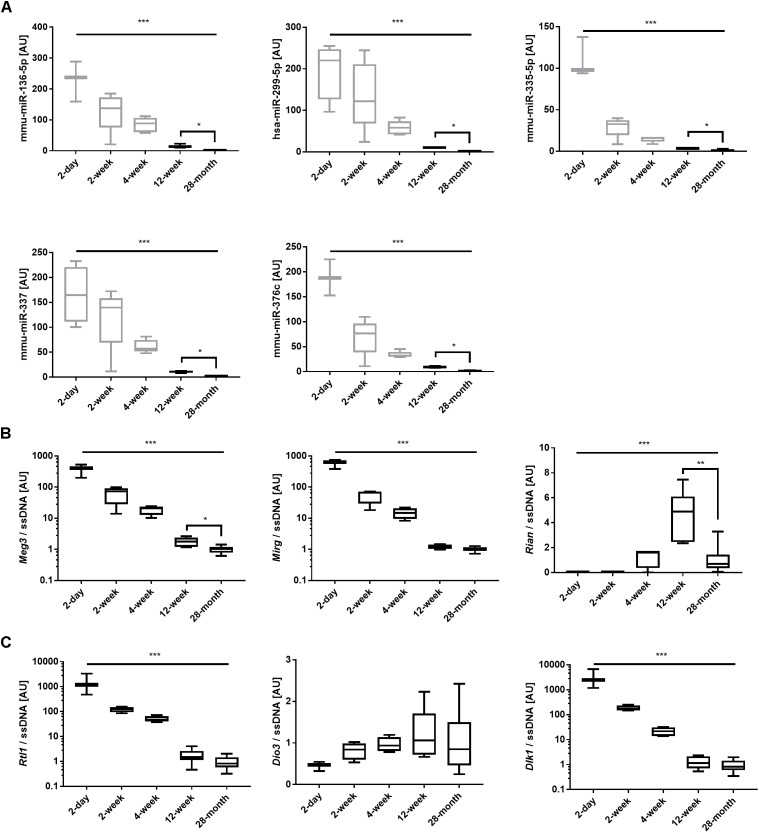
Expression levels of miRNAs, lncRNAs, and mRNAs from the Callipyge locus over the lifespan (5 ages). **(A)** Individual miRNA expression levels of mmu-miR-136-5p, mmu-miR-299-5p, mmu-miR-335-5p, mmu-miR-337, and mmu-miR-376c over the lifespan. The data are reported as Mean ± SEM. ^∗∗∗^, main effect of time, *p* < 0.001. The grayed data have been previously published in Lamon et al. (2017). **(B)** Individual expression levels of *Meg3, Mirg, Rian, Rtl1, Dio3*, and *Dlk1* over the lifespan. The data are reported as Mean ± SEM. ^∗∗∗^, main effect of time, *p* < 0.001. *Post hoc* tests: ^∗^*p* < 0.05 and ^∗∗^*p* < 0.01.

In a prior study, we reported the individual and collective expression levels of miRNAs (Lamon et al., 2017) and lncRNAs (Butchart et al., 2016) during the post-natal rapid growth phase of the quadriceps muscle, at 2 days, 2, 4, and 12 weeks after birth. MiRNAs were clustered using a robust quadratic model. These ages were then compared with the old muscle (aged 28 months). Out of the 13 miRNAs differentially regulated with aging, five miRNAs (mmu-miR-136-5p, mmu-miR-299-5p, mmu-miR-335-5p, mmu-miR-337, and mmu-miR-376c) were part of the two top clusters identified in Lamon et al. (2017). These clusters presented the largest changes over time and the highest biological significance, with “Cellular Development” and “Cellular Growth and Proliferation” being the highest ranked molecular and cellular functions associated with these clusters. The expression levels of these five miRNAs across the lifespan are depicted in Figure 5A. All five miRNAs not only presented a significant decrease with age (main effect, all *p* < 0.001) but also continued to decrease in old muscle after adjustment for multiple comparisons (all *p* < 0.05). Finally, the expression levels of *lncMyoD1, Neat_v1, Neat_v2, Malat1*, and *lincmd1* displayed significant differences with time, although without an aging-specific pattern (Supplementary Figure 1).

### Target Analysis

Target analysis identified three muscle-enriched genes that were potential targets of more than one miRNA of interest: *Myf6* (hsa-miR-299-5p, mmu-miR-434-3p, and mmu-miR-539-5p); *Pax7* (mmu-miR-136-5p and mmu-miR-539-5p); and *Mef2a* (mmu-miR-299-5p and mmu-miR-539-5p). The expression levels of these genes is shown in Supplementary Figure 2. *Pax7* and *Mef2a* mRNA levels significantly decreased with aging, while expression *Myf6* did not change, ruling out the hypothesis that the miRNAs identified in the screening may constitute primary negative regulators of their transcription.

### Gene and Protein Expression Levels of the Members of the *Dlk1-Dio3* Locus in Aging Quadriceps Muscle

Expression levels of the lncRNAs *Meg3, Mirg* and *Rian* presented significant changes over the lifespan (main effect, *p* < 0.001) (Figure 5B). *Meg3* and *Rian* displayed further significant decreases between 12-week and 28-month of age after adjustment for multiple comparisons (*p* < 0.05 and *p* < 0.01, respectively). The mRNA levels of *Rtl1* and *Dlk1* decreased by over 1000-fold between 2-days and 28-months of age (all *p* < 0.001), with no further reduction after the age of 12 weeks. *Dio3* expression did not vary at any time point (Figure 5C). In line with this result, DIO3 protein expression did not change with aging (Supplementary File S2 and Supplementary Figure 3A). The expression levels of the three other maternally expressed lncRNAs stemming from the mouse *Dlk1-Dio3* region (namely *anti-Rtl1, AK053394* and *AK050713*) was investigated. *Anti-Rtl1* was not detected in adult mouse muscle, whilst *AK053394* was expressed at levels that were hardly detectable by qPCR. In contrast, *AK050713* expression decreased by about 10-fold between age 2-week and 28-month (*p* < 0.001), with no further significant decreases between 12-week and 28-month of age (Supplementary Figure 3B).

## Discussion

MicroRNAs play a role in the regulation of skeletal muscle mass, metabolism and function over the lifespan and undergo high levels of regulation in skeletal muscle development and adaptation to health and disease conditions (Russell et al., 2013; Zacharewicz et al., 2014; Lamon et al., 2017; Butchart et al., 2018). More recently, the importance of lncRNAs in skeletal muscle growth and differentiation has started to emerge (Sunwoo et al., 2009; Cesana et al., 2011; Watts et al., 2013; Gong et al., 2015; Butchart et al., 2016). In two recently published studies, we investigated the expression patterns of 768 miRNAs and a series of selected lncRNAs in mouse quadriceps muscle between 2 days and 12 weeks of age (Butchart et al., 2016; Lamon et al., 2017). Following up on these results, the present study compared the expression levels of the same miRNAs and lncRNAs in mouse quadriceps muscle during early and very late (28 months) life stages. Out of the 12 miRNAs that were down-regulated with age, 10 clustered at the imprinted *Dlk1-Dio3* domain-containing locus, also known as ‘Callipyge,’ which controls important tissue homeostasis and pathogenesis processes (Benetatos et al., 2013). Coding (*Dlk1* and *Rtl1*) and non-coding (*Meg3, Mirg*, and *Rian*) members of the *Dlk1-Dio3* locus displayed 350-fold to 3500-fold decreases over the lifespan; however, only lncRNAs *Meg3* and *Rian* kept significantly decreasing in adulthood between the age of 12 weeks and 28 months.

Several studies have reported large-scale miRNA profiling in aging mouse skeletal muscle. In some studies, the data were not adjusted for multiple testing (Hamrick et al., 2010; Kim et al., 2014), therefore presenting a high false discovery rate (Ranganathan et al., 2016) and suggesting that the results must be interpreted with caution. One study used next-generation sequencing (NGS) and identified 23 miRNAs that were differentially expressed in 6-month and 24-month TA muscle following statistical adjustment and qPCR validation (Jung et al., 2017). Another recent study conducted a fluorescence based miRNA array in 3- and 26-month old gastrocnemius muscle and used qPCR to confirm the differential expression of 14 miRNAs with aging (Pardo et al., 2017). In line with previous studies, our preliminary analysis demonstrated that individual miRNA expression can be positively or negatively regulated with aging. Following statistical adjustment, significant changes reflected a trend toward a collective down-regulation of miRNA expression with aging. One miRNA target, mmu-miR-434-3p, was consistently downregulated in other miRNA profiling studies in aging muscle (Hamrick et al., 2010; Kim et al., 2014; Jung et al., 2017; Pardo et al., 2017). Mmu-miR-434-3p directly targets the anti-apoptotic eukaryotic translation initiation factor 5A1 (*Eif5a1*) 3′UTR, suppresses its expression in mouse primary myotubes and protects the myotubes from apoptosis (Pardo et al., 2017). This suggests that, *in vitro*, mmu-miR-434-3p downregulation may contribute to age-related muscle atrophy by alleviating repression of *Eif5a1* and allowing apoptotic processes to occur (Pardo et al., 2017). However, when observed in muscle tissue, apoptosis seems to mainly occur in the nuclei of interstitial cells, and whether these finding apply to whole muscle fibers *in vivo* has not been confirmed.

Ten of the 12 miRNAs that were significantly downregulated with aging clustered at the imprinted *Dlk1-Dio3* genomic region (Schmidt et al., 2000), suggesting that this miRNA cluster is expressed in a polycistronic manner. This is in line with the findings from a previous study that observed a number of miRNAs dysegulated in aging gastrocnemius muscle at the same chromosomic location (Kim et al., 2014). The *Dlk1-Dio3* genomic region, or ‘Callipyge,’ is located on human chromosome 14 and mouse chromosome 12. The Callipyge locus was originally identified on ovine chromosome 18 (Cockett et al., 1994), where a mutation in sheep resulted in pronounced hypertrophy of the hindquarters muscles. The name is derived from the Greek *calli*- (beautiful) and *pyge* (buttocks). This chromosomal region contains three paternally expressed imprinted genes: delta like non-canonical Notch ligand 1 (*Dlk1*), retrotransposon Gaglike 1 (*Rtl1*) and deiodinase, iodothyronine type III (*Dio3*), which are protein-coding; and four maternally expressed imprinted genes: maternally expressed 3 (*Meg3* or *Gtl2*), RNA imprinted and accumulated in nucleus (*Rian* or *Meg8*), *AK053394* and *AK050713*, which encode lncRNAs (Seitz et al., 2004; Glazov et al., 2008; Hagan et al., 2009). This locus also encodes *Mirg* (or *Meg9*), a large non-coding transcript that acts as a precursor for some of the 54 miRNAs (53 in the forward strand, 1 in the reverse strand) present in this region. This makes the Callipyge locus one of the largest miRNA clusters of the genome (Benetatos et al., 2013). In addition, an *anti-Rtl1* transcript stems from the non-coding strand and is processed into miRNA precursors that display perfect complementarity with *Rtl* (Seitz et al., 2003; Davis et al., 2005). This cluster of miRNAs is dysregulated in pathological conditions including a wide range of cancers (Benetatos et al., 2013). A single-base mutation in the intergenic region located between *Dlk1* and *Meg3* is responsible for the Callipyge trait (Freking et al., 2002), which, in sheep, translates to a 30–40% increase in muscle mass at the phenotypic level (Jackson et al., 1997). The Callipyge trait is only transmitted when the mutated allele comes from the father. This unusual, non-Mendelian mode of inheritance is termed ‘polar over dominance’ (Cockett et al., 1996; Georges et al., 2003). The homozygous Callipyge genotype does not present a strong muscle phenotype (Cockett et al., 1996; Georges et al., 2003), and it has been suggested that a negative acting maternally expressed effector, potentially *Mirg* or *anti-Rtl1*, suppresses the paternal expression of Dlk1 or Rtl1 (Georges et al., 2003). Mice lacking either of the paternally imprinted genes *Dlk1* (Moon et al., 2002), *Rtl1* (Sekita et al., 2008), or *Dio3* (Hernandez et al., 2006) display high rates of fetal deaths and/or severe growth defects. Interestingly, offspring born to mice lacking *Glt2* only survive if they carry the double-deletion, where normal expression of the ncRNAs of the locus is restored (Takahashi et al., 2009). Rather than acting as general, non-locus regulators of gene and protein expression, these ncRNAs may therefore play an intricate role in the maintenance of gene regulation at the *Dlk1-Dio3* locus itself.

Mouse muscle mass consistently decreases with aging (Barns et al., 2014; Soffe et al., 2016; White et al., 2016a,b), until the age of 24 months at least (Soffe et al., 2016). In our study, we observed large decreases in *Meg3, AK050713, Mirg, Rtl1*, and *Dlk1* expression over the lifespan. However, past the age of 12 weeks, there were only decreases in the expression levels of *Meg3* and *Rian*, suggesting the existence of upstream factors that individually regulate the transcription of the members of *Dlk1-Dio3* locus. The Callipyge phenotype is evident when the mutation is paternally inherited by a heterozygous animal and is associated with increases in transcript abundance of *Dlk1, Meg3, Rtl1*, and *Rian* in hypertrophied muscle (Bidwell et al., 2004). *Dlk1* and *Rtl1* expression is highest in the muscle of paternal heterozygous animals (Charlier et al., 2001; Bidwell et al., 2004), while *Meg3* and *Rian* transcripts are almost exclusively found in the maternal heterozygous and homozygous animals (Charlier et al., 2001; Bidwell et al., 2004). Protein-coding Dlk1 and Rtl1 are proposed effectors for muscle hypertrophy and are potentially regulated by a negative acting maternally expressed effector such as *Mirg* or *anti-Rtl1* (Georges et al., 2003). In line with this hypothesis, mice lacking the expression of the whole *Dlk1-Dio3* miRNA cluster display hypertrophy of the fast-twitch muscle and elevated expression of the Dlk1 protein (Gao et al., 2015). Indeed, *Dlk1* exclusively localizes in type II myofibers, which are significantly larger in sheep overexpressing *Dlk1* than in their corresponding WT controls (White et al., 2008). Mice lacking the negative regulator of muscle growth myostatin also display increased expression of *Dlk1, Meg3, Rian*, and *Mirg*, as well as a series of primary and mature forms of the miRNAs from the Callipyge locus (Hitachi and Tsuchida, 2017). The *Dlk1-Dio3* locus might therefore act a positive regulator of muscle growth that may be directly or indirectly regulated by myostatin. In contrast, *Dlk1* knockdown downregulated myosin heavy chain IIB gene expression, resulting in smaller muscles and reduced number of myofibers (Waddell et al., 2010). We therefore hypothesized that a downregulation of the *Dlk1-Dio3* locus may contribute to age-related muscle atrophy, providing a potential new mechanism for sarcopenia.

Surprisingly and despite a striking downregulation of the whole miRNA cluster, we only observed decreases in the maternally expressed imprinted genes *Meg3* and *Rian* at the age of 28 months when compared with 12 weeks, and no changes in the paternally expressed imprinted protein-coding genes. Specifically, there was no change in *Rtl1* or *Dlk1* gene expression with aging. MiR-127 and miR-136 are complementary to *Rtl1* mRNA (Davis et al., 2005) and suppressing their expression induces an increase in Rtl1 expression (Seitz et al., 2003); an effect that we did not observe in our study despite close to 10-fold decreases in the expression of these two miRNAs. Similarly, knocking out the imprinted *Dlk1-Dio3* miRNA cluster results in elevated levels of the Dlk1 protein and muscle hypertrophy, leading to the hypothesis that maternal expression of the miRNA cluster may regulate paternal expression of *Dlk1* (Gao et al., 2015). Again, and despite a strong collective dysregulation of at least 12 miRNAs of the *Dlk1-Dio3* cluster, we did not observe an effect on *Dlk1* gene expression levels. One possible explanation is that imprinting may not be rigorously maintained with age. For example, aging causes muscle transcriptome heterogeneity (Barns et al., 2014), which may result in partial loss of imprinting in some cells. The contribution of the *Dlk1-Dio3* locus to age-related muscle decline might therefore not be driven by the paternally imprinted *Rtl1* or *Dlk1*. Future analyses investigating the methylation status at the imprinting control element and other long-range regulatory elements within the locus may confirm how age affects the methylation status of these sites.

Mmu-miR-335-5p was the only downregulated miRNA that was not part of the *Dlk1-Dio3* genomic region. MiR-335 harbors within an intron of the *Mest* gene, which is paternally imprinted. While miR-335 is highly expressed during muscle development and regeneration, miR-335, unlike *Mest*, is not necessary to normal muscle growth (Hiramuki et al., 2015). Supporting an age-induced decrease of miR-335-5p expression, we previously showed that hsa-miR-335-5p was downregulated in aging human muscle (Zacharewicz et al., 2014). MiR-335-5p was up-regulated in myotonic dystrophy type 1 (DM1) muscle samples (Perbellini et al., 2011), in Duchenne Muscular Dystrophy (DMD) (Greco et al., 2009) and in several other neuromuscular disorders (Eisenberg et al., 2007), suggesting a potential role for miR-335-5p in muscle adaptation in extreme physiological conditions involving continuous degeneration/regeneration cycles. This is supported by several lines of evidence showing that miR-335-5p directly targets genes of the transforming growth factor-β (TGF-β) non-canonical pathways, such as mitogen-activated protein kinase 1 (MAPK1), resulting in reduced phosphorylation and inactivation of downstream pathway members (Lynch et al., 2012).

Finally, and despite significant changes during the early post-natal period confirming previous findings from our group (Butchart et al., 2016), changes in the expression levels of *lncMyoD1, Neat_v1, Neat_v2, Malat1*, and *Lincmd1* did not persist past the age of 12 weeks. These lncRNAs may therefore play an important regulatory role during hyperplasia and early hypertrophic growth and maturation, rather than in age-related muscle decline.

In conclusion, our study examined a combination of short and lncRNAs in old mouse skeletal muscle. We confirm that the collective decrease in muscle miRNA expression observed at early post-natal stages persists until old age, while muscle-specific lncRNAs display little regulation with aging. An unexpected finding was that the vast majority of the miRNA and lncRNA targets displaying significant decreases with aging were related to the Callipyge locus, which is associated with muscle development and hypertrophy. The dysregulation of this locus in aging muscle may therefore represent a potential new mechanism for age-related muscle decline.

## Availability of Data and Material

All data and material that are not presented in the main paper or supplementary files are available from the corresponding author on request.

## Author Contributions

SL, JM, and MG designed the study. SL and LB provided the samples. JM, KS, FG-R, and PDG collected the data. SL and SV analyzed the data and completed the statistical analysis. SL and MG funded the study. All authors contributed to manuscript writing and editing.

## Conflict of Interest Statement

The authors declare that the research was conducted in the absence of any commercial or financial relationships that could be construed as a potential conflict of interest.
