# Supplementary material for: MicroRNA and Long Non-coding RNA Regulation in Skeletal Muscle From Growth to Old Age Shows Striking Dysregulation of the Callipyge Locus
Source: Front Genet. 2018 Nov 16;9:548. doi: 10.3389/fgene.2018.00548 (PMC6250799; doi:10.3389/fgene.2018.00548)

RTL1 protein analysis (http://www.biorbyt.com/rtl1-antibody-1)

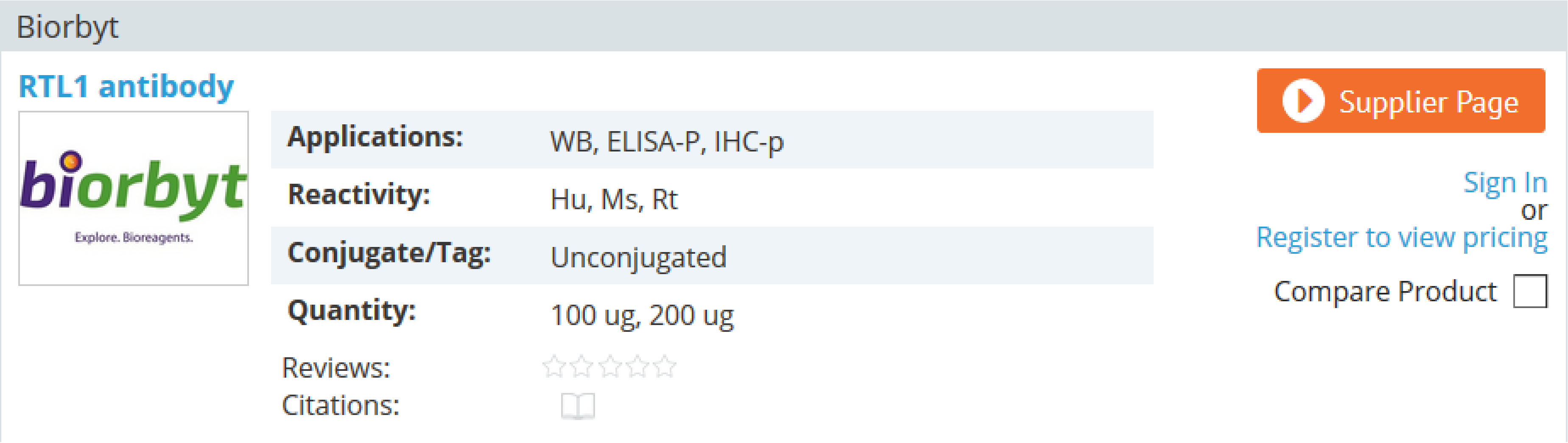

- This antibody is the only commercially available antibody targeting RTL1 in the mouse (source: www.biocompare.com)
- No WB example is provided on the antibody datasheet
- Predicted MW is 155 kDa
- No positive control is listed by the company

RTL1 protein analysis (http://www.biorbyt.com/rtl1-antibody-1) - **optimization**

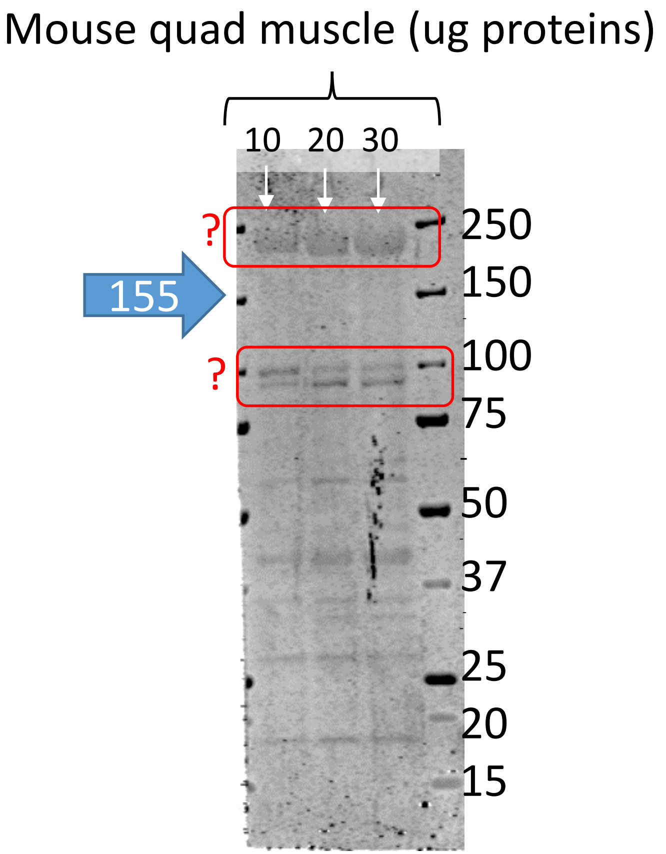

- This blot shows 10, 20 and 30ug of protein from mouse quadriceps muscle
- Primary antibody 1:500 diluted in 5% BSA in TBST
- TBST washes
- Secondary antibody 1:5000 diluted in 5% BSA in TBST
- TBS washes
- There is no band at, or around 155kDa (predicted molecular weight). Reports from other species in the literature consistently report a band at 150kDa +/- 10kDa, therefore we concluded that this antibody is non-specific

DLK1 protein analysis (https://www.cellsignal.com/products/primary-antibodies/dlk1-antibody/2069)

DLK1 Antibody #2069

PRINT

All / Category: Primary Antibodies / Products

APPLICATIONS	REACTIVITY	MW (kDa)	SOURCE
WB IP IHC IF F CHIP	H M	41	Rabbit

PREV NEXT

- This antibody is the gold-standard commercially available antibody targeting the mouse RTL1 protein (our laboratory has a standard protocol that has consistent success with Cell Signaling antibodies)
- COS cells transfected with mouse RTL1 is provided as a positive control on the antibody datasheet
- Predicted and observed MW is 41kDa

DLK1 protein analysis (https://www.cellsignal.com/products/primary-antibodies/dlk1-antibody/2069) - **optimization**

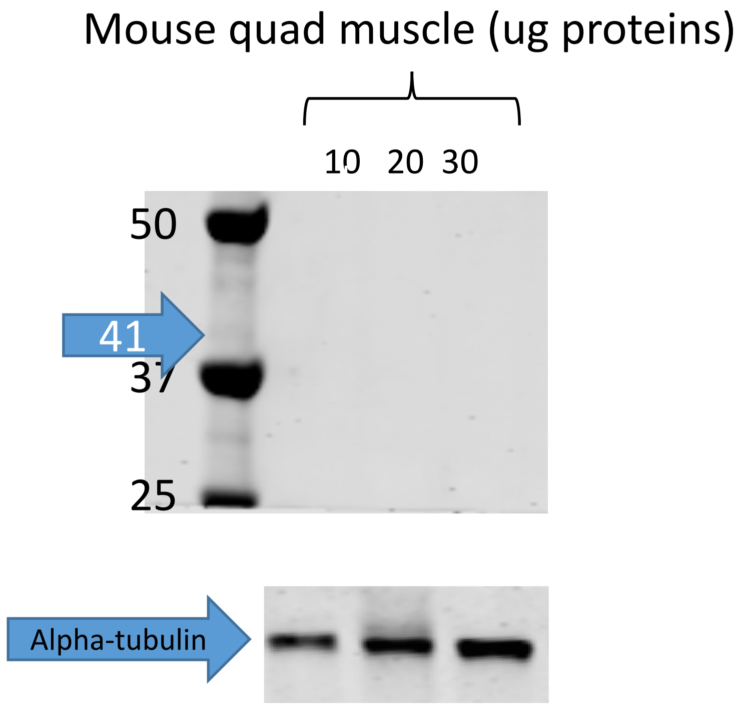

- This blot shows 10, 20 and 30ug of protein from mouse quadriceps muscle
- Primary antibody 1:250 diluted in 5% BSA in TBST
- TBST washes
- Secondary antibody 1:5000 diluted in 5% BSA in TBST
- TBS washes
- There is no band at, or around 41kDa (predicted and observed molecular weight). Information obtained from the company is that this antibody is not enough sensitive to pick up RTL1 endogenous expression
- As a positive control, the same blot was probed with alpha-tubulin (image below)

DIO3 protein analysis

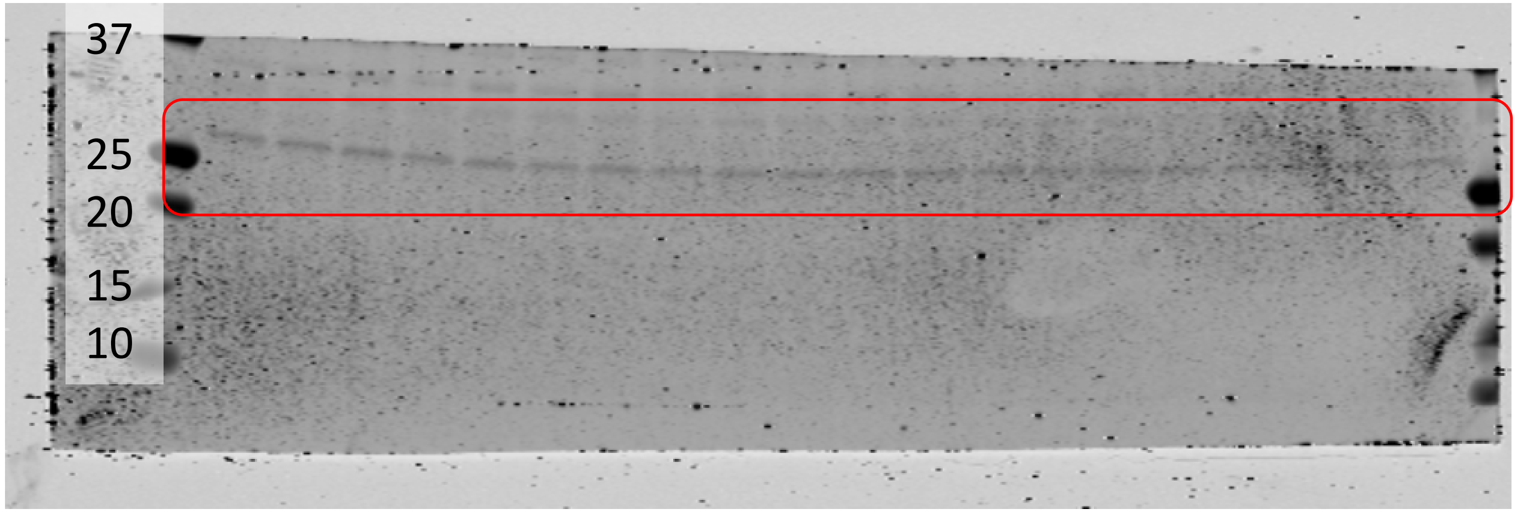

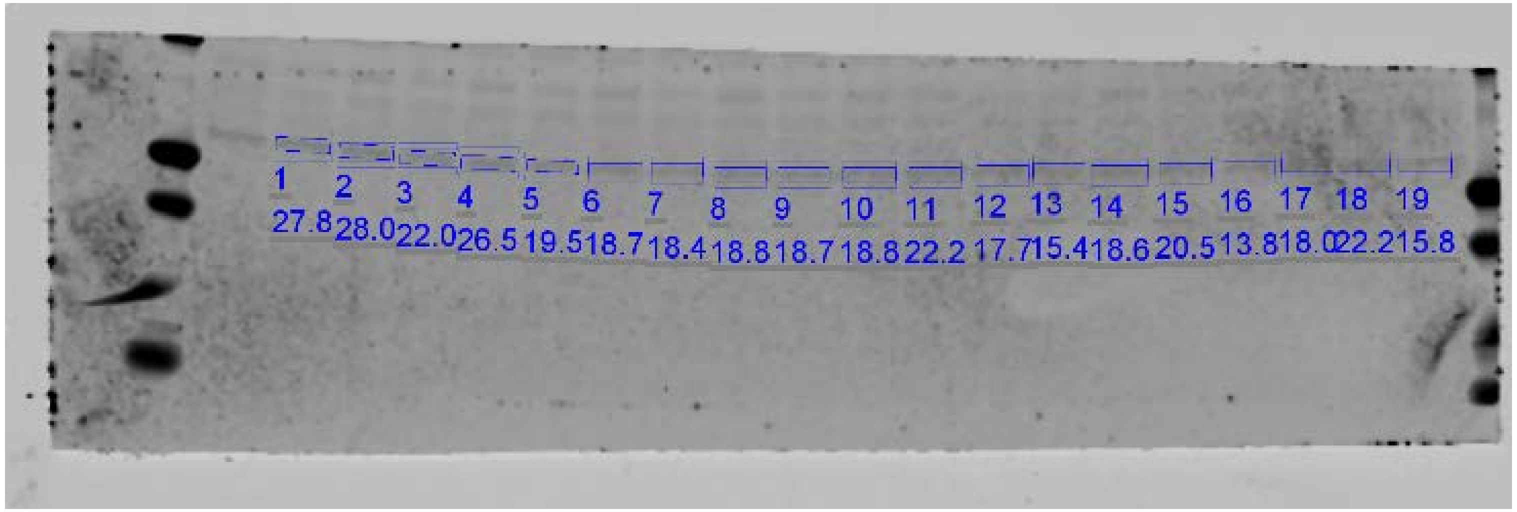

- This blot shows 20ug of protein from mouse quadriceps muscle samples
- Primary antibody 1:500 diluted in 5% BSA in TBST
- TBST washes
- Secondary antibody 1:5000 diluted in 5% BSA in TBST
- TBS washes
- Predicted and observed molecular weight: 31 kDa

DIO3 protein analysis – total protein quantification

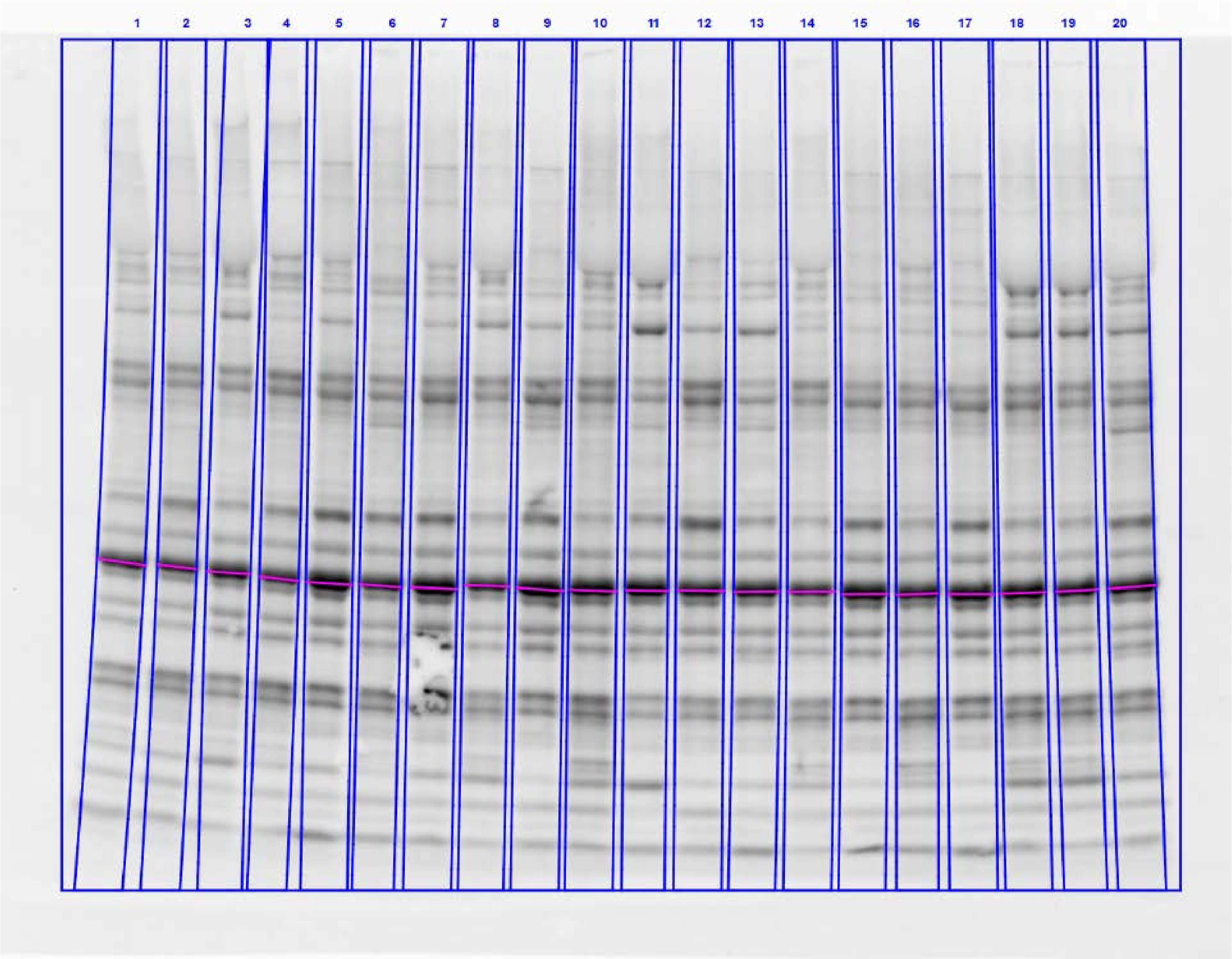

Supplement: Supplementary file 1 [file Data_Sheet_1.pdf]
