# Supplementary material for: MicroRNA and Long Non-coding RNA Regulation in Skeletal Muscle From Growth to Old Age Shows Striking Dysregulation of the Callipyge Locus
Source: Front Genet. 2018 Nov 16;9:548. doi: 10.3389/fgene.2018.00548 (PMC6250799; doi:10.3389/fgene.2018.00548)

Supplementary Material

**MicroRNA and long non-coding RNA regulation in skeletal muscle from growth to old age shows striking dysregulation of the Callipyge locus**

**Jasmine Mikovic^1^, Kate Sadler^1^, Lauren Butchart^2^, Sarah Voisin^3^, Frederico Gerlinger-Romero^1^, Paul Della Gatta^1^, Miranda Grounds^2^, and Séverine Lamon^1*^**

^1^Deakin University, School of Exercise and Nutrition Sciences, Institute for Physical Activity and Nutrition (IPAN), Geelong, Australia.

^2^The University of Western Australia, School of Human Sciences, Perth, Australia

^3^Victoria University, Institute of Health and Sport, Footscray, Australia

*** Corresponding author:**

Séverine Lamon, PhD, School of Exercise and Nutrition Sciences, Deakin University, Burwood 3125, Australia, Tel. (+61) 3 5227 8777, e-mail: [severine.lamon@deakin.edu.au](mailto:severine.lamon@deakin.edu.au)

**Supplementary Data 1.** Raw data from the miRNA array.

**Supplementary Figure 1.** Expression levels of muscle related lncRNAs over the lifespan. The data are reported as Mean ± SEM. *, p < 0.05; **, p < 0.01; ***, p < 0.001.


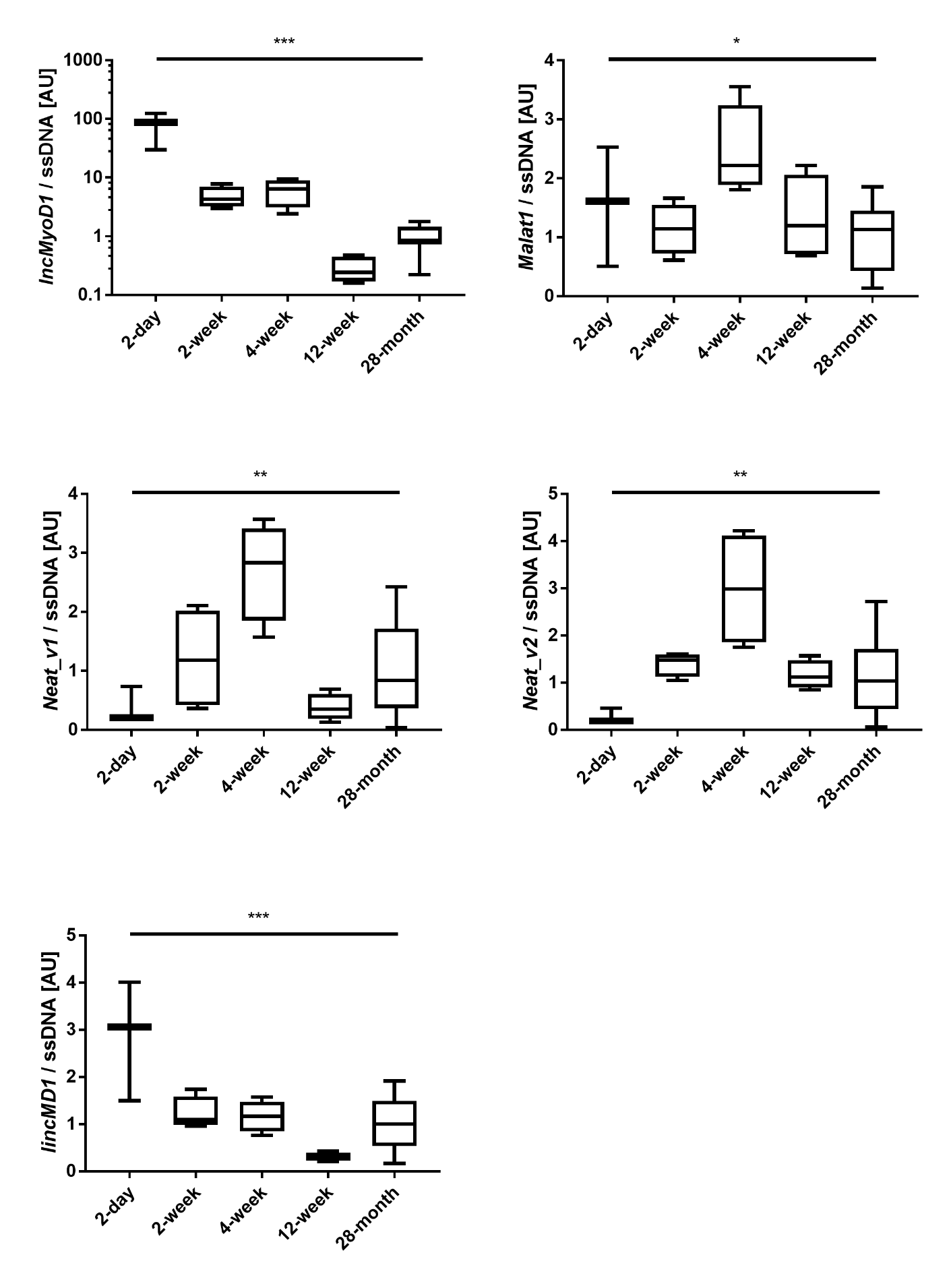


**Supplementary Figure 2.** Expression levels of putative target genes of the miRNAs of interest over the lifespan. The data are reported as Mean ± SEM. *, p < 0.05; ***, p < 0.001.

**Supplementary Figure 3. A)** Expression levels of the DIO3 protein in young and old muscle. **B)** Expression levels of *AK050713* over the lifespan. The data are reported as Mean ± SEM. ***, p < 0.001.


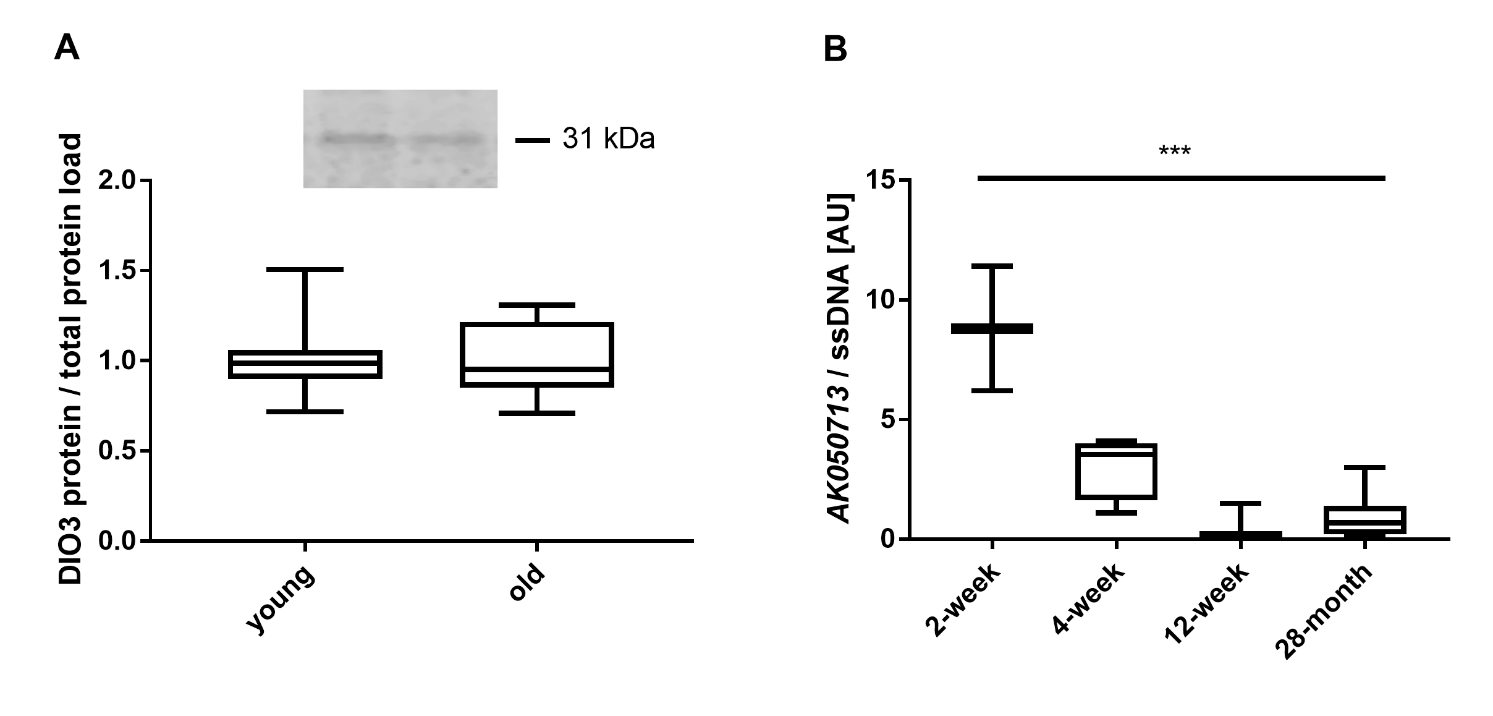

Supplement: Supplementary file 2 [file Data_Sheet_2.docx]
